# Sexual practices, their influencers, and utilization of HIV services among female sex workers in Mombasa County, Kenya

**DOI:** 10.11604/pamj.2024.47.209.41775

**Published:** 2024-04-24

**Authors:** Robert Abuga Mokinu, Shadrack Ayieko Yonge, Yves Lafort, Theodorus Gustavus Maria Sandfort, Joanne Ellen Mantell, Peter Bundi Gichangi

**Affiliations:** 1 County Government of Mombasa, Mombasa, Kenya,; 2 Department of Environment and Health Sciences, School of Applied and Health Sciences, Technical University of Mombasa, Mombasa, Kenya,; 3 Department of Uro-gynaecology, Ghent University, Ghent, Belgium,; 4 Department of Psychiatry, HIV Center for Clinical and Behavioral Studies at the New York State Psychiatric Institute, New York, USA,; 5 Columbia University Irving Medical Center, New York, USA,; 6 Technical University of Mombasa, Mombasa, Kenya; 7 Department of Public Health and Primary Care, Faculty of Medicine and Health Sciences, Ghent University, Ghent, Belgium

**Keywords:** Female sex workers, sexual practices, condom use, Kenya

## Abstract

**Introduction:**

female sex workers (FSWs) are vulnerable to acquiring HIV and other sexually transmitted infections due to unprotected sex. Understanding and addressing the gaps in safer sex among FSWs can help to reduce HIV acquisition and transmission. This study described sexual practices, their correlates and use of HIV services among FSWs in Mombasa County, Kenya.

**Methods:**

participants were recruited for a baseline survey by a time-location cluster randomized design at predetermined intervals from five bars and five clubs in Mombasa County until a sample size of 160 was reached. Descriptive statistics and inferential analysis using R were conducted, and p<0.05 was regarded as statistically significant.

**Results:**

nearly all (99%) of the participants were unmarried, and 11% had tertiary education. Ninety-eight percent (98%) reported vaginal intercourse, 51% reported using alcohol/drugs before sex, and 28% practiced unprotected intercourse. About 64% had tested for HIV within three months, 14% believed that it is safe to reuse condoms, and 10% that it is safe to engage in unprotected sex. In bi-variate analysis, FSWs were more likely to engage in unprotected intercourse if they reported more frequent sex, more frequent sex with regular clients, poor HIV knowledge, alcohol/drug use, and violence. In multivariate analysis, risky sexual practices were associated with frequency of sexual intercourse, alcohol/drug use, and poor HIV knowledge.

**Conclusion:**

female sex workers engage in unprotected sex while under the influence of substances, belief in re-using condoms and have high frequency of sexual intercourse. Inadequate knowledge of HIV and substance use significantly correlated with unprotected sex. Interventions to address these modifiable factors are needed to mitigate the risk of HIV among FSWs.

## Introduction

Female sex workers (FSWs) are considered among the key populations for contracting human immunodeficiency virus (HIV) and other sexually transmitted infections (STIs) as a result of risky sex practices which refer to sex without a condom [1-3]. Sex work can involve provision of sexual services under the guise of entertainment and recreational enterprises [4] or with the clear objective of exchanging sex for money or goods [5]. The risk of acquiring HIV infection among FSWs globally is estimated to be 13 times higher than in the general population of women [6-8]. Sex work often takes place in the context of substance use [9-12] which may increase FSWs´ likelihood of unprotected sex and hence, high risk of contracting HIV [13,14]. In a systematic review/meta-analysis of two studies, 15.7% of FSWs practice anal intercourse which is more risk of contracting HIV than vaginal intercourse if unprotected [3,15]. In Africa, most FSWs are young, economically disadvantaged, lack stable housing, have low education and use alcohol/drugs, making them vulnerable to a range of health and social problems [16]. Routine HIV testing among FSWs is often low due to difficulty in accessing the services as a result of discrimination, especially in Africa [17,18]. Studies have shown that some FSWs engage in unprotected sexual intercourse, use condoms inconsistently or incorrectly [8,19], engage in sex work at a young age and engage in anal intercourse with multiple sexual partners [20].

Adolescent girls who engage in sex work [21] are more vulnerable to HIV infection than older women due to lack of skills to negotiate condom use [22]. Additionally, they are at risk for health issues brought on by early or unexpected pregnancies [23]. In sub-Saharan Africa, FSWs serve as a bridge for HIV transmission to the general population [24,25]. They also have higher HIV prevalence (3 times higher) compared to the general population of women. Risky sexual practices are elevated by low social status, low education level, alcohol use and violence [10,26]. In a national surveillance study in Kenya in 2022, HIV prevalence among FSWs was 29.3% [27] contributing 14.0% to the national prevalence which is 4.8% [28]. The HIV prevention programs have not adequately addressed the needs of FSWs to ensure their easy access to HIV testing, prevention, treatment, and human rights protection [29]. The confluence of substance abuse, violence, social discrimination, low socioeconomic status and negative perception towards safer sex exacerbate FSWs´ vulnerability to HIV/STIs [30]. In Mombasa, the site of this study, FSWs operate from hotels, clubs, bars, streets and private homes [31]; approximately 19.9% of FSWs engage in sex work before age 18, have low educational level, are unmarried and financially constrained [32]. Only 88.0% of them report using a condom at last sex with a client. With an occasional non-client it was 73% and with a regular partner 62% [33]. Cases of condom breakage are associated with improper use; male partners´ dislike using condoms, hence intentionally tamper with them during use [3,1]. This study assessed sexual practices, determinants of unprotected sex, HIV knowledge and use of HIV testing services among FSWs in Mombasa County, Kenya, to inform policy and planning for promotion of safer sex practices to mitigate HIV acquisition and transmission.

## Methods

**Study design:** this study used a time-location cluster randomized design. Female sex workers participated in a baseline survey prior to implementation of project boresha (Improve), a pilot, multilevel, and structural HIV risk-reduction intervention for female and male sex workers and their male clients. Project Boresha provided on-site peer education on HIV risk-reduction strategies, condom use and lubricants and sexual health services, including HIV and STI testing, counseling and care in bars and clubs in Mombasa, Kenya. Data were collected between December 2014-May 2015.

**Study settings and population:** the study was conducted in Mombasa County which has a population of 1,433,689 (52% male and 48% female) covering 229.9 square kilometers [3,4]. Among FSWs in Mombasa, HIV prevalence is 12.1% [3,5]. Participants were FSWs residing in Mombasa County who were 18 years and older, willing to consent, visibly sober and had sexual intercourse in exchange for money or goods with a client in the last one month.

**Independent variables:** these included age, educational level, current marital status, economic status (level of income per month in Kshs.), alcohol/drug use, frequency of sexual intercourse (number of times FSWs had vaginal or anal sex for money/goods in past 30 days), violence and knowledge about HIV.

**Outcome variable:** the variable of interest in this study was risky sexual practice, defined as engaging in vaginal or anal intercourse without the use of a condom at some point during sex work in the last one month.

**Sample size determination:** a sample size of 160 was calculated using the formula for cross-sectional studies with power of 80, 95% CI with two-tail 5% margin of error to detect a 12% rate of condom-protected sex among FSWs in Mombasa.

**Data collection:** data collection was done at three purposively selected pairs of bars and clubs with different characteristics, from which FSWs usually operate. Peer educators approached FSWs entering the venue during the identified time period. Recruitment continued until the target sample size of 160 FSWs was obtained. Interviews were conducted face-to-face in discreet spaces at each of the selected venues. Only study staff and potential participants were permitted access to the space. Counselling was offered as well as provision of referral services.

**Data management and statistical methods:** data was collected on a tablet using Open Data Kit (ODK) software. Verification (checking the data's accuracy, completeness and consistency of data) was conducted. Data were exported to R data analysis software for analysis. Descriptive analysis was performed. Inferential statistics were computed using Pearson’s Chi-square tests. Factors associated with unprotected sex were initially assessed using bivariate regression, and those that were significant at the p<0.05 level were tested for their independent association with the outcome using multiple logistic regression model. Age, economic position, educational status, and length of time working as a sex worker were all considered potential confounders. A backward, stepwise approach was used to eliminate variables that were not statistically significant. Bootstrapping was too used to address high correlation.

**Ethical considerations:** ethical approval and clearance were obtained from the Kenyatta National Hospital and University of Nairobi Ethics Review Committee (Protocol #P548/09/2014) and the New York State Psychiatric Institute/Columbia University Department of Psychiatry (Protocol #6842) prior to data collection. A research permit was obtained from National Commission for Science, Technology and Innovation. Participation was voluntary. Their names were kept confidential, and the information gathered was used solely for the purposes of the study. Written informed consent was obtained from participants prior to the interviews.

## Results

**Socio-demographic characteristics:** at the onset of the data collection, the target number of respondents was 160 however, one respondent did not take part in the interview session until the end. Of the 159 FSWs enrolled in this study, the mean age of was 30.23±9.97 years, median age 30 years, lower quartile 25 years, upper quartile 35 years. Almost a half (46%) had completed primary education and 11% tertiary education. Most participants resided in Mombasa County (82.4%) and the majority (94%) were unmarried (Table 1).

**Table 1:** social-demographic characteristics, sexual practices, HIV knowledge and HIV testing among female sex workers

Variable	Frequency (n) (N=159)	Percentage (%)
**Demographic characteristics**		
**Age**		
19-24	38	24
25-30	47	30
Above 30	74	47
**Education**		
None	7	4
Some primary education	73	46
Some secondary education	62	39
Tertiary	17	11
**Marital status**		
Not married	149	94
Married	10	6
**Residence**		
Mombasa County	131	82
Kilifi County	28	18
**Sexual practices**		
**Age at first sex in exchange for money/goods**		
Below 18 years	27	17
18-25 years	91	57
Above 25 years	41	26
**Times exchanged sex for money/goods in past 30 days**		
≤10 times and below	32	20
>10 times	127	80
**Number of times engaged in sexual intercourse with paying sexual partners (regular clients) in the past one month**		
<5 times	39	25
>5 times	120	75
**Number of times engaged in vaginal sexual intercourse with paying sexual partners (regular clients) in the past 30 days**		
<5 times	37	23
>5 times	122	77
**Number of times engaged in anal sexual intercourse at least once with paying sexual partners (regular clients) in the past 30 days**		
0	150	94
≥1	9	6
**Paying sexual partners (regular clients) used alcohol or drugs before sex**		
Yes	118	74
No	41	26

**Sexual practices:** nearly three-fifths (57%) of participants engaged in sex for money/goods when they were between the ages of 18 and 25 years, and 17% engaged in sex work before the age of 18. Most (80%) reported that they had exchanged sex for money/goods more than 10 times in the past 30 days. Three-quarters (75%) of participants had engaged in sexual intercourse with paying sexual partners (paying) more than five times in the past one month. Among those who had engaged in sexual intercourse with paying sexual partners, 77% reported engaging in vaginal intercourse more than five times in the past month with paying sexual partners. The majority (94%) indicated that they had not engaged in anal sexual intercourse with paying sexual partners during the same time period. Slightly more than two-fifths (43%) of FSWs reported they receive between USD5-10 from a male client in exchange for sex. Risky sex was practiced by 28% of study participants (Table 1).

**HIV testing and knowledge:** nearly all participants (99%) reported they had ever tested for HIV, but only 64% had tested less than three months ago. Fourteen percent believed that it is safe to reuse condoms. Additionally, 10% of FSWs agreed that it is safe to have sex without a condom. A few participants (3%) were of the opinion that as long as both partners wash themselves after sex, it is not necessary to use condoms and 9% agreed that if someone has HIV, it can be seen straightaway (Table 1.1).

**Table 1.1:** social-demographic characteristics, sexual practices, HIV knowledge and HIV testing among female sex workers

Participants used alcohol or drugs before sex with paying sexual partners (regular clients)	Frequency (n) (N=159)	Percentage (%)
Yes	63	47
No	96	53
**Number of times physically hurt by someone while doing sex work or because of being a sex worker in the past 12 months**		
0	109	69
>1	50	31
**Number of times forced to have sex or sexually assaulted or raped by someone while doing sex work or because of being a sex worker in the past 12 months**		
0	121	70
≥1	38	24
**Amount of money (Ksh.) received for having sex**		
Below 500	47	30
500- 1000	69	43
Above 1000	43	27
**HIV knowledge**		
**Belief that it is safe to use a condom more than once**		
True	22	14
False	137	86
**Belief that it is safe to have sex without a condom if with regular partner**		
True	16	10
False	143	90
**Belief that as long as both partners wash themselves after sex, it is not necessary to use condoms**		
True	5	3
False	154	97
**Belief that if someone has HIV, it can be seen straightaway**		
True	15	9
False	144	91
**HIV testing**		
**Ever tested for HIV**		
Yes	157	99
No	2	1
**When last tested for HIV**		
<3 months ago	102	64
3 to 6 months ago	31	20
>6 months ago	26	16
sample size, n-count		

**Substance use and violence:** almost a half (47%) of FSWs reported having used alcohol or drugs before sex at least once with a client. Physical violence while doing sex work or because of being a sex worker in the past 12 months was reported by 31% of the FSWs and 24% reported being sexually assaulted or raped at least once in the past 12 months (Table 2).

**Table 2:** characteristics associated with risky sex practices among female sex workers

Variable	Bi-variate		Multivariate	
	COR (95% CI)	p-value	aOR (95% CI)	p-value
**Age**				
19-24	Ref		-	-
25-30	0.87(0.31- 2.42)	0.7950		
Above 30	1.64(0.68-4.00)	0.2815		
**Marital status** Married	Ref	-	-	-
Not married	1.13 (0.28-4.58)	0.8427		
**First age got money/goods for sex Below 18 years**	Ref			
18-25 years	1.49(0.51-4.38)	0.4911		
Above 25 years	2.82(0.89-8.95)	0.0799		
**Number of times had sex for money/goods times in past month**				
10 times and below	Ref	-	Ref	0.000*
Above 10	4.99(2.20-11.35)	0.0001*	4.99(2.22-11.54)	
**Number of times engaged in sex with paying sexual partners (regular clients) in the past month**				
Below 5 times	Ref	-	Ref	-
More than 5 times	3.27(1.19-9.02)	0.0148	3.27(1.28-10.12)	0.022*
**Number of times engaged in vaginal sexual intercourse with regular paying**				
sexual partners in the past month	Ref	-	Ref	-
Less than 5	3.01(1.09-8.31)	0.0254	3.01(1.17-	0.034*
More than 5			9.32	
**Number of times engaged in anal sex with regular paying sexual partners in the past month**				
0	Ref	-		
Once or more	0.73(0.15-3.68)	0.7568		
**Paying sexual partners (regular clients) used alcohol or drugs before sex**				
Yes	Ref	-		
No	0.45(0.18-1.11)	0.0780		
**Participant used alcohol or drugs before sex with paying sexual partners (regular clients)**				
Yes	0.06 (0.02-0.14)	0.0001	0.06(0.02-0.13)	p<0.0
No	Ref	-	-	00*
**Number of times physically hurt by someone while doing sex work or because of being a sex worker in the past one month**				
0	1.18(0.56-2.48)	0.6570		
At least once or more times	Ref	-		
**Number of times forced to have sex or raped by someone while doing sex work or because of being a sex worker**				
0	Ref	-		
At least once or more times	1.28(0.58-2.84)	0.5398		
**Believe it is safe to use a condom more than once**				
True	2.52(1.00-6.36)	0.0496		-
False	Ref	-		
**Believe it is safe to have sex without a condom if it is with regular partner**				
True	2.97(1.04-8.50)	0.0502 -	2.97(1.02-8.65)	0.042* -
False	Ref		Ref	
**Ever tested for HIV**				
Yes	0.78	0.3787		
No	Ref	-	-	
**When last tested for HIV**				
Less than 3 months ago	0.61(0.24-1.55)	0.3925		
3 up to 6 months ago	0.47(0.13-1.68)	0.2648		
More than 6 months	Ref			

cOR: crude odds ratio; aOR"adjusted odds ratio; Ref:reference; *Uncorrected Chi-square

**Characteristics associated with risky sex: bivariate analysis:** female sex workers who had sex (vaginal/anal) more than 10 times in the past 30 days were most likely to have engaged in unprotected sex compared to those who had sex 10 times or less during this time period (cOR=4.99, 95% CI: 2.20-1.35) and this difference was statistically significant (p=0.000). Those who engaged in sexual intercourse with regular paying sexual partners more than five times in the past one month had 3.27 higher odds of engaging in unprotected sex compared to their counterparts who had sex less than five times within the same period (cOR=3.27, 95% CI: 1.19-9.02; p=0.015). Participants who engaged in vaginal intercourse with paying sexual partners more than five times in the past one month were 3.01 times (cOR=3.01, 95% CI: 1.09-8.31; p=0.025) more likely to practice unprotected sex than those who had vaginal intercourse less than five times in the past one month. Female sex workers who used alcohol or drugs before sex with regular paying sexual partners were 0.06 times (cOR=0.06, 95% CI: 0.02-0.14: p=0.000) more likely to practice unprotected sex too than those who did not use alcohol or drugs before sex with same partners. Those who believed that it was safe to use a condom more than once and those who believed that it was safe to have sex without a condom if it was with regular partner were 2.52 times (cOR=2.52, 95%: CI: 1.00-6.36: p=0.049) and 2.97 times (cOR=2.97, 95%: CI: 1.04-8.50 p=0.050) more likely to engage in sex without using condoms than those with contrary opinions (Table 3).

**Independent predictors of risky sex practices:** variables that were significant in the bi-variate analysis were subjected to multivariate logistic regression analysis to determine independent predictors of risky sex practices. Variables independently associated with unprotected sex were: having sex (vaginal and anal) more than 10 times in past one month (adjusted odds' ratio [aOR]=4.99, 95% CI=2.22-11.54, p=0.000), engaging in sexual intercourse (vaginal and anal) with paying sexual partners (regular clients) more than five times in the past one month (AOR=3.27, 95% CI: 1.28-10.12, p=0.022), having used alcohol or drugs before sex with paying sexual partners (regular clients) (aOR=0.06, 95% CI: 0.02-0.13, p=0.000) and having an opinion that it was safe to have sex without a condom if it was with regular partners (aOR=2.97, 95% CI: 1.02-8.65, p=0.042) (Table 3).

## Discussion

Like in this study, 94% of participants in a study conducted by Roberts *et al*. [26] among young women who sell sex in Mombasa, Kenya, were not married. Unmarried women who engage in sex work to earn a living for their families often find themselves engaging in unsafe sexual practices due to financial constraints. However, in this study, unprotected sex was higher among married than unmarried FSWs, married FSWs perceive themselves to be at less risk. These findings were aligned with a 2020 study conducted by Putra and Januraga [4] among indirect FSWs in Bali, Indonesia, and a 2022 Beksinska *et al*. [3,6] among FSWs in Nairobi, Kenya. About 17% began their careers in the sex industry before turning 18 years, similar to the findings of another study conducted in Mombasa, which found that about 20% of FSWs initiate sex work before 18 years of age [31]. Adolescent girls engaging in transactional sex are far more susceptible to sexual assault, HIV/STI and limited access to healthcare services than older ones, as reported in a study in Kampala, Uganda [3,7]. In this study, the prevalence of FSWs engaging in unprotected sex with clients was about 28% contrary to 19.6% reported by Bitty Anderson *et al*. [30] in Togo. We found out that vaginal sex was more prevalent (99%) than anal sex (1%) which was similar to the 2020 findings of studies conducted among FSWs in Eswatini and among adolescent girls and young women in Mombasa, Kenya, by Owen *et al*. and Bhattacharjee *et al*. [3,22]. The majority (80%) had sex more than ten times in the past one month. Female sex workers who engaged in sex for money/goods more frequently (more than 10 times within a month) were more likely to have practiced unprotected sex compared to those who did so less frequently. Also, results demonstrated that FSWs engaging in sex more frequently with paying sexual partners (>5 times in a month) were more likely to practice risky sex than those who had sex less frequently with paying partners. Our study findings differed from those of Mueses- Marín *et al*. [3,8] on the frequency of sex acts and consistency of condom use among FSWs who found out that those FSWs who perceived themselves to be at high risk of HIV infection use condoms more frequently than those who do not. Again, our findings indicated that FSWs engaging in vaginal sexual intercourse more frequently (more than five times in a month) with regular paying sexual partners were more likely to practice unprotected sex compared to those with less than five encounters with same sexual partners.

Nearly a third (32%) of participants had encountered violence from their intimate sexual partners, and almost a quarter (24%) experienced sexual assault. In this study, 47% of FSWs reported having used alcohol or drugs prior to having sex with their paying sexual partners. These findings were nearly the same as those in a study by Beksinska *et al*. [3,6] in Nairobi, Kenya, which showed that 42% of FSWs used alcohol or other drugs before sex. The study findings demonstrated that when FSWs used alcohol or drugs before sex with their regular male clients, the risk for unsafe sex was much higher than when they didn´t use alcohol or drugs before sex with the same clients. Previous studies by Bradburn *et al*. and Lancaster *et al*. [31,39] in Mombasa, Kenya, and in Lilongwe, Malawi, reported similar findings. According to Leddya *et al*. [40] in Tanzania, FSWs who are intoxicated are more likely to experience gender-based violence, usually when both (they and their partners) are intoxicated. This study assessed HIV knowledge among FSWs with a focus on condom use, beliefs in the safety of reusing condoms and having sex without condoms with regular partners. Believing that it is safe to have sex without condoms with regular partners was associated with increased risk of unsafe sex. These findings were consistent with those of Putra and Januraga [4] in Bali, Indonesia, who found that FSWs with sufficient knowledge of HIV were likely to use condoms correctly and consistently and did not succumb to the pressures of male clients who preferred condomless sex.

The majority (99%) of FSWs had ever tested for HIV but only 64% had tested within the last three months as recommended by National AIDS and STI Control Program (NASCOP) in the Kenyan HIV Prevention and Treatment Guidelines, 2022. These findings were consistent with Ochonye *et al*. and Mutagoma *et al*. in Nigeria and Rwanda respectively that many FSWs engage in high-risk sex behaviors such as unprotected sex, have sex while drunk and having many sexual partners but rarely test for HIV [41,42]. Almost 14% believed in reusing condoms and nearly 10% were of the opinion that if someone has HIV it can be seen straightaway, highlighting the knowledge gap among FSWs about HIV despite previous efforts in creating awareness.

**Strengths and limitations:** major strengths of this study include; random representative sample that was produced as a result of sampling techniques used for data gathering among targeted FSWs, Recruitment of participants was conducted from the settings where FSWs and clients arrange sexual encounters, trained research assistants assisted in data collection. This study had the following limitations; the study was cross-sectional. The study focused only on FSWs and not their sexual partners, who are likely to influence their sexual practices.

## Conclusion

The study results indicated that FSWs are engaging in unprotected sex while under influence of alcohol/drugs and having high frequency of sexual intercourse. Few FSWs had tested for HIV within the last three months: FSWs are as recommended to test for HIV after every three months (NASCOP, 2022). Underage sex work is still a problem. Female sex workers who engaged in sex more frequently were less likely to use condoms consistently. Interventions to address these modifiable factors such as creating FSWs’ social networks are needed to control and prevent HIV among them; social networking among FSWs build peer solidarity/trust and may contribute greatly to condom utilization, which is one of the key interventions together with pre-exposure prophylaxis used in the fight against HIV/AIDS.

### What is known about this topic

- *Female sex workers carry the highest burden of HIV and STIs due to risky sexual practices which include unprotected sex, sex while intoxicated, belief that it is safe to re-use of condoms and high frequency of sexual intercourse with their male partners who dislike using condoms;*
- *Female sex workers are a vulnerable population at risk of HIV acquisition and onward HIV transmission to the general population;*
- *Many FSWs lack power to negotiate condom use, due to their financial situation and male clients’ offer to pay more for unprotected sex*.

### What this study adds

- *This study assessed sexual practices that are unsafe, with the aim of increasing awareness and uptake of safer sexual practices among FSWs; according to the study, FSWs with high exposure risks use condoms less frequently than their peers with lower exposure;*
- *This study identified determinants of risky sexual practices; the effectiveness of safer sex is challenged by the socioeconomic status of the FSWs, alcohol use and inadequate knowledge on safer sex;*
- *This study found out that controlling alcohol/drugs use before sex among FSWs lowers the risk of unprotected sex; sex work is practiced by all women regardless of education level*.
